# Supplementary material for: Tpo1-mediated spermine and spermidine export controls cell cycle delay and times antioxidant protein expression during the oxidative stress response
Source: EMBO Rep. 2013 Oct 18;14(12):1113–9. doi: 10.1038/embor.2013.165 (PMC3981086; doi:10.1038/embor.2013.165)
Supplement: Supplementary Information [file embor2013165s1.pdf]

SUPPLEMENTARY INFORMATION

Krueger et al.

Tpo1-mediated spermine and spermidine export controls cell cycle delay and times antioxidant protein expression during the oxidative stress response.

Yeast strain generation

Yeast strains used in this study were derived from the gene deletion project [1], are based on the S288c derivative BY4741 [2], and have been generated as described below (Table 1).

For the generation of the *TPO1* overexpressing strain, *TPO1* was amplified from BY4741 genomic DNA using primers *TPO1*-*Bam*HI-Fwd (AGGGATCCATGTCGGATCATTCTCCCAT) and *TPO1*-*Xho*I-Rev (AGCTCGAGAGCGGCGTAAGCATACTTGG). The PCR product was digested with *Bam*HI and *Xho*I and ligated into the respective sites of plasmid pRS303GPD, a pRS303 vector containing the promoter of glycerol 3-phosphate dehydrogenase 1 (*GPD1*). The construct was validated by re-sequencing.

Table 1: Yeast strain genotypes

Strain name in manuscript	genotype	parent strain	Strain generation and modifications
**Wild type**	*MAT*a (*his3*Δ1::*HIS3) leu2*Δ0 *met15*Δ0 *ura3*Δ0	BY4741	To achieve isogenity between the yeast strains compared in this study, *the HIS3* locus in BY4741 was reconstituted by integration of empty pRS303GPD
Δtpo1	*MAT*a (*his3*Δ1::*HIS3) leu2*Δ0 *met15*Δ0 *ura3*Δ0 *tpo1*Δ::*kanMX*4	Euroscarf Y01516 (BY4741 Δ*tpo1*)	*The HIS3* locus in the *tpo1*Δ strain of the deletion project was reconstituted by integration of empty pRS303GPD
**oex*TPO1***	*MAT*a *his3*Δ1::*HIS3-GPD1pr-TPO1 leu2*Δ0 *met15*Δ0 *ura3*Δ0	BY4741	*TPO1* placed under control of the *GPD1* promoter was integrated into the *HIS3* locus of BY4741 using pRS303GPD
**BY4741**	*MAT*a *his3*Δ1 *leu2*Δ0 *met15*Δ0 *ura3*Δ0	S288c	*[2]*
BY4741 Δtpo1	*MAT*a *his3*Δ1 *leu2*Δ0 *met15*Δ0 *ura3*Δ0 *tpo1*Δ::*kanMX*4	BY4741	*[1]* Euroscarf Y01516

Screening the MATa gene deletion collection.

For screening the haploid (*MAT*a) gene deletion collection for altered oxidant response, all strains were grown overnight in 96-well plates, subsequently diluted and inoculated in fresh yeast extract peptone dextrose (YPD) media. Then, they were shaken at 900 rpm and 30 °C (Heidolph Titramax 1000 with Heidolph Incubator 1000) until mid-log phase, in which media was supplemented with 1.25 mM H_2O_2 (H1009; Sigma-Aldrich). Growth and recovery of the strains were monitored photometrically using a microplate reader (SpectraMax 250 Microplate reader, Molecular Devices). The screen was conducted four times and candidate strains were identified by calculating the growth rate difference between 30 and 90 minutes after H_2O_2 treatment.

The 15 identified candidate strains were deleted for YBR101C, YBR276C, YDR462W, YGL127C, YGR259C, YIL047C, YIL054W, YIL066C, YIL093C, YJR087W, YLL028W, YMR066W, YMR158W, YPL230W or YPL253C. These candidates were validated by a) oxidant spot testing to exclude generally oxidant-resistant strains, b) mating assays with BY4742 (its *MAT*alpha counterpart [2]) to exclude potential contaminants of other genotypes, c) resembling the auxotrophy of the parent library (BY4741) background and d) complementing their growth phenotype by re-introducing the deleted gene via plasmid transformation.

Spot testing.

H_2O_2 spotting tests (Fig 1B,C) were conducted as previously described [3]. In brief, yeast strains were pre-grown overnight, diluted to an OD_{600} of 0.20 in complex (YPD) media and grown exponentially. Then strains were spotted at a series of 10-fold dilutions onto synthetic complete (SC) agar plates containing H_2O_2 with or without 1 mM spermidine (S4139; Sigma-Aldrich, St. Louis, MO) or spermine (S4264; Sigma-Aldrich) and incubated at 30 °C for 2 days.

Growth assays.

For growth assays, yeast strains were pre-grown overnight, diluted to an OD_{600} of 0.15 in SC (Fig 1A, Fig 3B-D and Fig 4) or YPD (Fig S3) media and distributed on 96-well microtiter plates. Growth at 30 °C was recorded for at least 4 replicates on a multimode detector (DTX 880, Beckman Coulter). H_2O_2 and/or spermine treatments were conducted in the exponential growth phase at the indicated time points. Data analysis and calculation of growth parameters were conducted using R package grofit (model free spline fit) as described [4].

Determination of polyamine concentrations.

Yeast cells were harvested by centrifugation (5,000 g, 5 min), washed with 1 ml synthetic complete (SC) media, and the pellet snap-frozen on dry ice. Then, 210 mg (±5 mg) glass beads (500-625 µ) were added, the cells were re-suspended in 800 µl $HClO_4$ (0.2 M), and lysed through rigorous shaking (2 * 20 sec, 6.5 m/s) at 4 °C on a FastPrep-24 instrument (MP Biomedicals). Then, cell debris was removed by centrifugation (12,000 g for 10 min at 4 °C), 500 µl of the supernatants were collected and neutralised with 3 M sodium carbonate (50 µl).

Polyamines were quantified by liquid chromatography/tandem mass spectrometry after dansylation. In brief, a yeast cell extract was derivatised with dansylic acid as described [5], and separated on a Zorbax Eclipse Plus C_{18} (RRHD, 2.1 x 50 mm, 1.8 µm) column on an Agilent 1290 HPLC instrument. Quantification was carried out on a triple quadrupole mass spectrometer (Agilent 6460), monitoring MRM transition 423 m/z - 170 m/z for dansyl-spermidine, 568 m/z - 170 m/z for dansyl-spermine, and 555 m/z - 220 m/z for dansyl-putrescine. The method was controlled for derivatisation efficiency, stability and linear range.

SWATH-MS.

Targeted proteomics was conducted with minor modifications as described previously [6]. Strains were grown to mid-exponential phase, and treated with 1.5 mM H_2O_2. 30 ml of the yeast cultures corresponding to ~30 OD was sampled, washed, and tryptic peptides were prepared according to the RapidACN protocol [7]. Data was acquired on an AB/Sciex TripleTOF5600 mass spectrometer coupled to an Eksigent nanoLC 2D-ultra operating in SWATH mode, setting the SWATH m/z acquisition window to 25 Da. Automatic extraction of product ion spectra was performed using Spectronaut software (Biognosys) monitoring 8 transitions per peptide, and allowing only peptides with a Qvalue < 0.01. Analysis of proteins abundance and clustering was conducted in Expander [8]. For targeted SWATH, selected proteotypic peptides were manually extracted from SWATH data with the help of Skyline software [9, 10] monitoring the peptides and transitions listed in Table 2. The abundance of each peptide was normalised to the first time point, and overall protein abundance was calculated by averaging a minimum of 4 peptides. Protein abundance was then normalised to the corresponding expression of the GAPDH isozyme Tdh1, which served as internal reference control.

Table 2: Transitions used for targeted SWATH-MS.

protein	*tryptic peptide*	*precursor m/z*	*product m/z*	*product ion*	*product charge*
**SSA1**	TTPSFVAFTDTER	736.3	938.5	y8	1
			839.4	y7	1
			768.4	y6	1
	NQAAMNPSNTVFDAK	804.3	978.5	y9	1
			1092.5	y10	1
			630.3	b6	1
	NFNDPEVQADMK	704.3	917.4	y8	1
			1146.5	y10	1
			1032.5	y9	1
	VNDAVVTVPAYFNDSQR	947.9	1097.5	y9	1
			499.3	b5	1
			400.2	b4	1
**HSP104**	ALTILTLAQK	536.3	673.4	y6	1
			887.6	y8	1
			560.3	y5	1
	GADTNTPLEYLSK	704.9	849.5	y7	1
			1064.6	y9	1
			950.5	y8	1
	VIGATTNNEYR	619.3	1025.5	y9	1
			897.4	y7	1
			796.4	y6	1
	ISSIVIFNK	510.8	820.5	y7	1
			620.4	y5	1
			907.5	y8	1
**HSP82/HSC82**	ELISNASDALDK	638.3	920.4	y9	1
			1033.5	y10	1
			833.4	y8	1
	QLETEPDLFIR	680.9	760.4	y6	1
			1119.6	y9	1
			990.5	y8	1
	AELINNLGTIAK	628.9	830.5	y8	1
			943.6	y9	1
			1056.6	y10	1
	NPSDITQEEYNAFYK	909.9	1063.5	y8	1
			1191.5	y9	1
			414.2	b4	1
	SISNDWEDPLYVK	783.4	619.4	y5	1
			1049.5	y8	1
			863.5	y7	1
**ENO1**	IGSEVYHNLK	580.3	674.4	y5	1
			1046.5	y9	1
			773.4	y6	1
	KAADALLLK	471.8	814.5	y8	1
			743.5	y7	1
			683.4	b7	1
	AADALLLK	407.8	672.4	y6	1
			743.5	y7	1
			557.4	y5	1
	VNQIGTLSESIK	644.9	834.5	y8	1
			947.5	y9	1
			1075.6	y10	1

Flow cytometry.

Flow cytometry was performed on a Becton Dickinson FACS Aria II SORP. Strains were pre-grown overnight, diluted to an OD_{600} of 0.15 in YPD, and cultivated at 30 °C for 4.5 hours. Then, cells were treated with 0.75 mM H_2O_2 (Fig 3A) or 1.25 mM H_2O_2 (Fig S2) for 150 minutes, or left untreated, and fixed with 70 % EtOH (ice cold) rotating overnight at 4 °C. RNA was digested by 0.1 mg/ml RNase A in 50 mM sodium citrate (pH 7.0) overnight at 37 °C. Samples were stained with 32 μg/ml propidium iodide in 50 mM sodium citrate for 1 hour at RT in the dark. For each sample more than 100.000 cells were analysed with FlowJo 9.4.11 software.

H_2O_2 quantification.

For the determination of the anti-oxidant properties of polyamines (Fig S4), SC medium was mixed with 1.5 mM H_2O_2, and 2 mM spermidine or spermine. After 30 min incubation at 30 °C, free H_2O_2 concentrations were determined using the Amplex Red Hydrogen Peroxide/Peroxidase Kit (Invitrogen, Carlsbad, CA) according to manufacturer's instructions.

SUPPLEMENTARY FIGURES

Fig S1

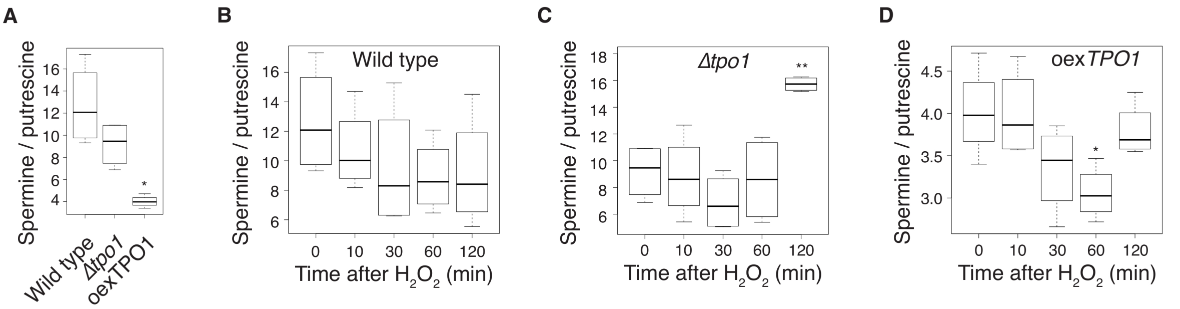

Spermine in *TPO1* mutants and during the oxidative stress response.

(**A-D**) Intracellular ratio of spermine to putrescine, which is not Tpo1 substrate, estimated from LC-MS/MS measurements. Wild type, Δ*tpo1* and *TPO1* overexpressing (oexTPO1) cells were grown exponentially in SC, treated with 1.5 mM H_2O_2 and sampled at indicated time points after treatment. Error bars represent SD (n = 4); Student's *t*-test: * = $p \leq 0.05$, ** = $p \leq 0.01$.

(**A**) *TPO1 overexpression decreases basal spermine level.* Spermine/putrescine ratio in wild type and *TPO1* mutant cells before H_2O_2 addition.

(**B**) *Spermine levels in wild type cells tend to decline during H_2O_2 treatment.* Spermine/putrescine ratio in H_2O_2-treated wild type.

(**C**) *TPO1 deletion leads to spermine accumulation during H_2O_2 treatment.* As in **B**, but with Δ*tpo1* cells.

(**D**) *TPO1 overexpression reduces spermine levels during H_2O_2 treatment.* As in **B**, but with oex*TPO1* cells.

Fig S2

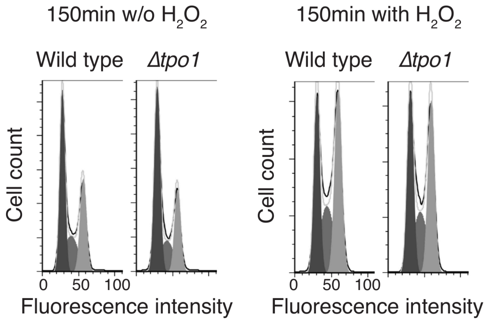

H_2O_2-treated wild type and *Δtpo1* cells arrest similarly in the G2 phase of the cell cycle.

This figure corresponds to Fig 3a, but with another H_2O_2 concentration. Wild type and Δ*tpo1* cells were grown exponentially in YPD, then, treated with 1.25 mM H_2O_2 or left untreated for 150 min, sampled and stained with propidium iodide. (Left and middle panel) Cell cycle distribution of at least 100.000 cells was measured by FACS and analysed using FlowJo 9.4.11 software. (Right panel) Relative increase in G2 over G1 cells. Error bars represent SD (n = 3).

Fig S3

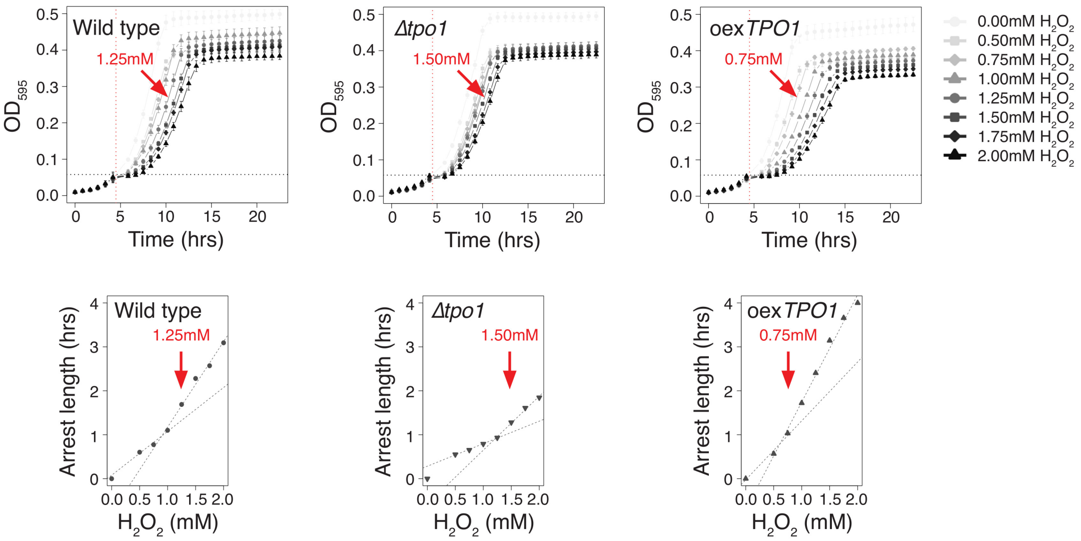

Rich media partially restores a cell cycle arrest extension in *Δtpo1* cells

Wild type, Δ*tpo1* and *TPO1* overexpressing (oexTPO1) cells were exponentially grown in yeast extract peptone dextrose (YPD) medium and treated with incremental sub-lethal H_2O_2 doses. (Upper panels) Growth curves as determined photometrically. (Lower panels) Correlation plot of arrest length and H_2O_2 concentration. Arrows indicate the H_2O_2 threshold concentration inducing the prolonged cell cycle arrest. Error bars represent SD (n = 4).

Fig S4

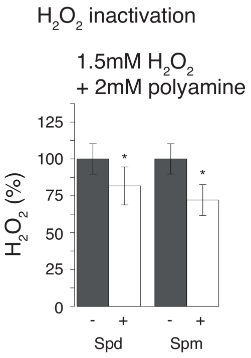

Spermidine and Spermine partially deplete H_2O_2.

1.5 mM H_2O_2 was incubated in vitro with 2 mM spermidine (Spd) or spermine (Spm) at 30 °C for 30 min. H_2O_2 concentrations were determined enzymatically as described in the methods section. Error bars represent SD (n = 3) Student's *t*-test: * = $p \leq 0.05$, ** = $p \leq 0.01$.

Fig S5

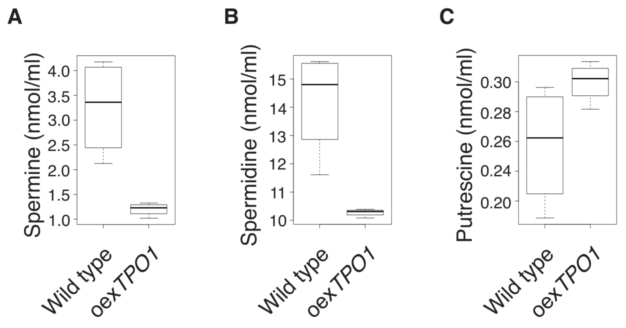

Non-normalised, absolute levels of spermine, spermidine and putrescine as determined from whole cell extracts of wild-type and *TPO1* overexpressing mutants as determined by LC-MS/MS. A clear concentration decline in spermine and spermidine upon overexpression of *TPO1* is detected.

Supplemental References

1. Winzeler EA *et al* (1999) Functional characterization of the S. cerevisiae genome by gene deletion and parallel analysis. *Science* **285:** 901-906
2. Brachmann CB, Davies A, Cost GJ, Caputo E, Li J, Hieter P, Boeke JD (1998) Designer deletion strains derived from Saccharomyces cerevisiae S288C: a useful set of strains and plasmids for PCR-mediated gene disruption and other applications. *Yeast* **14:** 115-132
3. Ralser M *et al* (2007) Dynamic rerouting of the carbohydrate flux is key to counteracting oxidative stress. *J Biol* **6:** 10
4. Kahm M, Hasenbrink G, Lichtenberg-Fraté H, Ludwig J, Kschischo M (2010) grofit: Fitting Biological Growth Curves with R. *Journal of Statistical Software* **33:**
5. Reyes-Becerril M, Esteban MA, Tovar-Ramirez D, Ascencio-Valle F (2011) Polyamine determination in different strains of the yeast Debaryomyces hansenii by high pressure liquid chromatography. *Food Chemistry* **127:** 1862-1865
6. Gillet LC, Navarro P, Tate S, Roest H, Selevsek N, Reiter L, Bonner R, Aebersold R (2012) Targeted data extraction of the MS/MS spectra generated by data independent acquisition: a new concept for consistent and accurate proteome analysis. *Mol Cell Proteomics*
7. Bluemlein K, Ralser M (2011) Monitoring protein expression in whole-cell extracts by targeted label- and standard-free LC-MS/MS. *Nature Protocols* **6:**
8. Shamir R, Maron-Katz A, Tanay A, Linhart C, Steinfeld I, Sharan R, Shiloh Y, Elkon R (2005) EXPANDER--an integrative program suite for microarray data analysis. *BMC Bioinformatics* **6:** 232
9. MacLean B *et al* (2010) Skyline: an open source document editor for creating and analyzing targeted proteomics experiments. *Bioinformatics* **26:** 966-968
10. Abbatiello SE *et al* (2013) Design, Implementation, and Multi-Site Evaluation of a System Suitability Protocol for the Quantitative Assessment of Instrument Performance in LC-MRM-MS. *Mol Cell Proteomics*
